# Impact of HSV-1 Infection on Alzheimer’s Disease Neurodegeneration Markers: Insights from LUHMES 2D and 3D Neuronal Models

**DOI:** 10.3390/ijms27020642

**Published:** 2026-01-08

**Authors:** María Martín-Rico, Blanca Salgado, Inés Beamonte, Isabel Sastre, María J. Bullido, Jesús Aldudo

**Affiliations:** 1 Centro de Biologia Molecular Severo Ochoa (CBM), CSIC—Universidad Autonoma de Madrid, 28049 Madrid, Spain; maria.martin@cbm.csic.es (M.M.-R.); bsalgado@cbm.csic.es (B.S.); inesbp04@gmail.com (I.B.); isastre@cbm.csic.es (I.S.); 2 CIBER de Enfermedades Neurodegenerativas, Instituto de Salud Carlos III, 28031 Madrid, Spain; 3 Instituto de Investigacion Sanitaria del Hospital Universitario La Paz—IdiPAZ, 28046 Madrid, Spain

**Keywords:** HSV-1, Alzheimer’s disease, LUHMES cells, 3D neuronal cultures, beta-amyloid, phosphorylated tau, neurodegeneration, lysosomal alterations

## Abstract

Herpes simplex virus type 1 (HSV-1) has been proposed as an environmental risk factor for Alzheimer’s disease (AD). Viral infection of neuronal cells can reproduce hallmark pathological features of AD, including intracellular beta-amyloid (Aβ) accumulation, tau hyperphosphorylation, and lysosomal dysfunction. However, the molecular mechanisms underlying these alterations remain unclear, partly due to limitations of existing experimental models. Here, we established both two-dimensional (2D) and three-dimensional (3D) LUHMES neuronal cultures—a human mesencephalic-derived neural cell line that differentiates rapidly into mature neurons—to investigate HSV-1-induced AD-associated markers. Our results demonstrate that HSV-1 infection induces key features of AD, including intracellular accumulation of Aβ peptides and hyperphosphorylation of tau protein. Moreover, we observed disruptions in the autophagy–lysosome pathway, characterized by increased LC3-II levels, reduced cathepsin activity, and impaired lysosomal burden. Notably, these AD-like alterations were reproduced in 3D LUHMES neuronal aggregates, confirming their susceptibility to productive HSV-1 infection. Collectively, these findings indicate that HSV-1 not only triggers AD-like neuropathological markers but also disrupts cellular clearance mechanisms that may contribute to neuronal dysfunction and degeneration. This study validates the 3D LUHMES system as a useful human neuronal model to study virus-induced neurodegeneration and its mechanistic links to AD pathology.

## 1. Introduction

Alzheimer’s disease (AD) is the most common cause of dementia, characterized by progressive neurodegeneration and cognitive impairment [1]. Despite extensive research efforts, its exact etiology remains elusive, and current therapeutic strategies only provide limited symptomatic relief [2]. Although senile plaques—primarily composed of beta-amyloid (Aβ) peptides—and neurofibrillary tangles (NFTs)—formed by hyperphosphorylated tau protein—have been recognized as key features of AD for over a century [3], the molecular mechanisms driving the progression of the disorder are still not fully understood. Historically, the amyloid cascade hypothesis has dominated AD research, proposing that accumulation of Aβ peptides initiates a cascade of pathological events culminating in neuronal death [4]. However, accumulating evidence suggests that multiple factors, including infectious agents, significantly contribute to the complexity and progression of AD pathology [5].

Herpes simplex virus type 1 (HSV-1) is a highly prevalent human pathogen that infects a large proportion of the global population, typically during early life [6]. It is primarily associated with orolabial lesions but has the capacity to infect neurons and establish lifelong latency within the peripheral nervous system, particularly in sensory ganglia. Periodic reactivation of the virus can lead to recurrent symptoms and viral shedding [7]. Beyond its classical clinical presentations, growing evidence suggests that HSV-1 may exert long-term effects on the central nervous system (CNS). The virus is neurotropic and capable of invading the brain, where it may persist in a latent or low-grade replicative state [8]. Reactivation or chronic infection in the CNS has been implicated in various neurological disorders, including encephalitis, and more recently, neurodegenerative diseases such as AD [9,10]. In this context, understanding HSV-1’s mechanisms of neuroinvasion, latency, reactivation, and the cellular pathways it disrupts—particularly in neuronal cells—is essential for elucidating its potential role in neurodegeneration.

A growing number of studies suggest a possible association between chronic HSV-1 infection and an increased risk of developing AD. Epidemiological studies have consistently identified associations between HSV-1 and a higher risk of AD, particularly among individuals carrying the APOE-ε4 allele [11,12,13,14]. Neuropathological analyses have revealed that HSV-1 DNA is frequently detected in the brains of AD patients, especially in brain regions severely affected by the disease, i.e., the hippocampus/limbic system [15]. Furthermore, experimental studies demonstrate that HSV-1 infection can directly induce hallmark AD features, such as Aβ accumulation [16], tau hyperphosphorylation [17], neuroinflammation [18] and synaptic dysfunction [19]. However, the detailed molecular and cellular mechanisms linking HSV-1 infection to AD pathology remain to be fully elucidated.

Emerging evidence also highlights the critical involvement of the autophagy–lysosomal system in both AD pathology and viral infections [20,21]. In AD, impaired autophagic flux and lysosomal dysfunction lead to the accumulation of toxic protein aggregates, including Aβ and phosphorylated tau, thereby contributing to neuronal stress and degeneration [20,22]. Likewise, several neurotropic viruses—including HSV-1—are known to interfere with autophagy and lysosomal pathways to favor their replication and persistence within host cells [21,23]. Thus, disruption of the autophagy–lysosomal system represents a convergent mechanism linking HSV-1 infection to AD-like neurodegenerative processes.

Advanced cellular models capable of recapitulating key aspects of human brain physiology offer invaluable tools for investigating virus-induced neurodegenerative processes. LUHMES (Lund human mesencephalic) is a human dopaminergic neuronal progenitor cell line derived from embryonic mesencephalic precursor cells, conditionally immortalized through the integration of the *v-myc* oncogene controlled by a tetracycline-regulated system. These cells exhibit a remarkable capacity for proliferation and, upon controlled suppression of the oncogene, differentiate rapidly into mature neurons that display physiological and morphological properties characteristic of the human nervous system [24]. Importantly, LUHMES cells differentiate exclusively into post-mitotic neurons under these conditions. Numerous foundational studies have demonstrated that differentiated LUHMES cultures form highly homogeneous neuronal populations, and do not give rise to glial cells of any lineage, including astrocytes, oligodendrocytes or microglia [24,25,26]. A key advantage of the LUHMES cellular model is its ability to maintain homogeneous and stable neuronal differentiation in long-term cultures, providing consistent cell lines for reproducible studies. This makes them particularly valuable for large-scale pharmacological screening assays and investigations into genetic and environmental factors involved in neurodegenerative diseases. Additionally, LUHMES cells are suitable for modeling neurological disorders in both two-dimensional (2D) and three-dimensional (3D) culture systems [25,27,28], enabling the generation of models that more closely replicate the spatial and cellular complexity of the human brain environment. Furthermore, these cells have been successfully used to study the effects of infectious and toxic agents on neuronal physiology [29,30], thereby facilitating an understanding of the mechanisms underlying the onset and progression of CNS pathologies. To date, only a few studies have explored HSV-1 infection in LUHMES cultures, and most have focused on the establishment and maintenance of viral latency in 2D cultures [30,31,32]. The effects of productive lytic infection on AD-related markers have not been characterized in this model.

Considering the above-mentioned advantages of LUHMES cells, mainly their growing capacity and ability to differentiate into a homogeneous neuronal phenotype, the present study aimed to develop and characterize a 3D LUHMES-based model of HSV-1 infection to investigate the molecular mechanisms leading to virus-induced neurodegeneration. We demonstrate that HSV-1 infection in this system reproduces AD-like alterations, including intracellular Aβ accumulation, tau hyperphosphorylation, and lysosomal dysfunction. Collectively, these findings provide new insights into the potential role of HSV-1 in AD pathogenesis and establish a human neuronal platform suitable for mechanistic studies to dissect the interplay between viral infection and neurodegeneration.

## 2. Results

### 2.1. LUHMES Cells Efficiently Differentiate into a Homogeneous Neuronal Population

**Figure 1:**
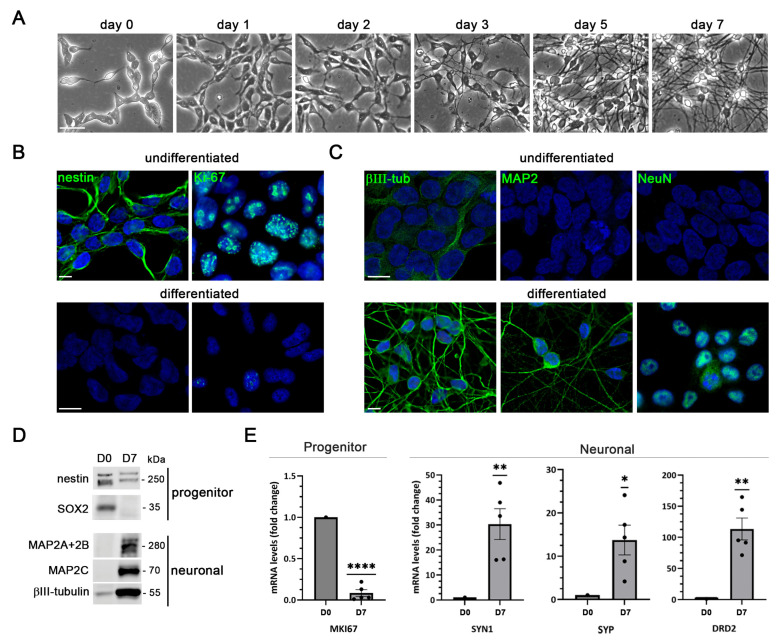
Differentiation of LUHMES cells into post-mitotic neurons in 2D cultures. (**A**) Representative phase-contrast images of LUHMES cells at different days of differentiation. Scale bar: 50 µm. (**B**) Immunofluorescence staining of the proliferation marker Ki-67 and the neural progenitor marker nestin in undifferentiated and 7-day-differentiated LUHMES cells. Scale bar: 10 µm. (**C**) Immunofluorescence images of neuronal markers (βIII-tubulin (βIII-tub), microtubule-associated protein 2 (MAP2) and neuronal nuclei antigen (NeuN) in undifferentiated and 7-day-differentiated LUHMES cells. DAPI-stained nuclei are also shown. Scale bar: 10 µm. (**D**) Western blot analysis of neural progenitor (nestin and SOX2) and neuronal (βIII-tub and MAP2 isoforms) marker levels in undifferentiated (D0) and 7-day-differentiated (D7) LUHMES cells. (**E**) Analysis of gene expression of proliferation (*MKI67*) and neuronal (synapsin I (*SYN1*), synaptophysin (*SYP*) and D2 dopamine receptor (*DRD2*)) markers by reverse transcription followed by quantitative PCR (RT-qPCR) in undifferentiated (D0) and 7-day differentiated cells (D7). Graph data show the mean ± standard error of the mean (SEM) of 5 independent experiments (*n* = 5) (one-sample *t*-test; * *p* < 0.05; ** *p* < 0.01; **** *p* < 0.0001).

LUHMES cells are a human neuronal precursor cell line derived from embryonic mesencephalon that can be terminally differentiated into post-mitotic dopaminergic-like neurons under defined culture conditions. In their proliferative state, LUHMES cells exhibit a compact and rounded morphology. As reported in earlier studies, withdrawal of growth factors combined with tetracycline exposure induces rapid neuronal differentiation, with neurite outgrowth visible within 2–3 days and a pronounced neuronal morphology by day 5. By this time, cells form dense neuronal networks clearly observable under phase-contrast microscopy (Figure 1A).

Immunocytochemical analysis revealed that proliferating LUHMES cells express neural progenitor and proliferation markers, including nestin—a neuroepithelial stem cell marker involved in cytoskeletal organization—and Ki-67, a proliferation marker associated with DNA replication (Figure 1B). Upon differentiation, these markers were downregulated, whereas the expression of mature neuronal markers such as βIII-tubulin, microtubule-associated protein 2 (MAP2) and neuronal nuclei antigen (NeuN) was strongly upregulated (Figure 1C). Unlike other neural stem cell models, LUHMES cells do not give rise to glial lineages such as astrocytes or oligodendrocytes, highlighting their suitability for studies focused exclusively on neuronal biology.

Protein and gene expression analysis by Western blot and reverse transcription followed by quantitative PCR (RT-qPCR) further confirmed the transition from a progenitor to a neuronal phenotype. A marked decrease in nestin, SOX2—a transcription factor essential for embryonic development—and Ki-67 (*MKI67*) was accompanied by strong upregulation of neuronal markers including βIII-tubulin, MAP2, synapsin 1 (*SYN1*), synaptophysin (*SYP*), and D2 dopamine receptor (*DRD2*) (Figure 1D, E). Overall, these findings demonstrate the rapid and efficient neuronal differentiation of LUHMES cells into a homogeneous population of post-mitotic human neurons, providing a robust in vitro system to investigate cellular and molecular mechanisms underlying neurodegeneration.

### 2.2. LUHMES Cells Are Permissive to HSV-1 Infection in Both Proliferative and Differentiated States

**Figure 2:**
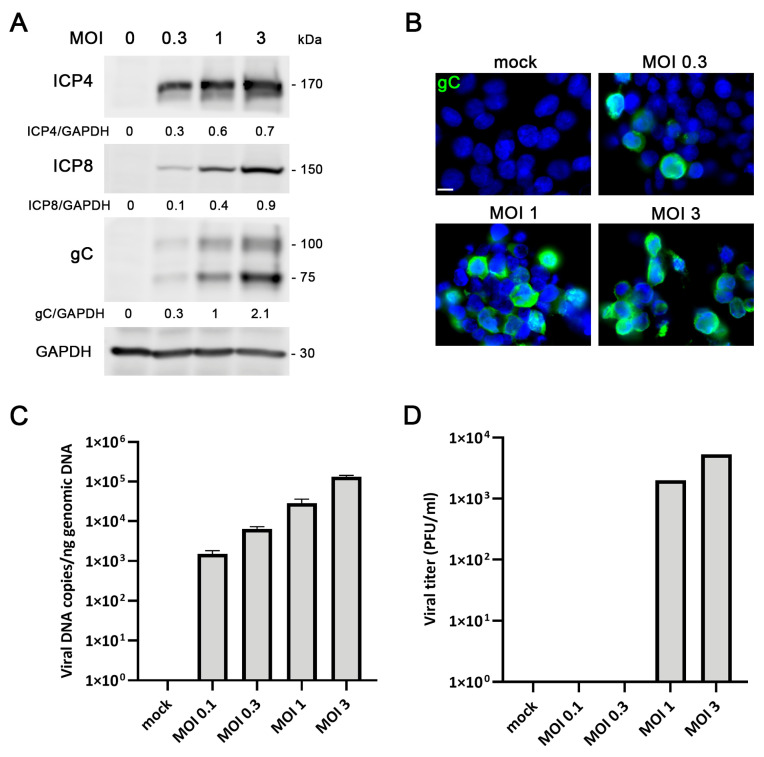
Characterization of Herpes simplex virus type 1 (HSV-1) infection in proliferative LUHMES cells in 2D cultures. (**A**–**D**) Proliferative LUHMES cells were uninfected (mock) or infected with HSV-1 at different multiplicities of infection (MOI) for 18 h (hpi), and the infection efficiency was monitored. (**A**) Analysis of ICP4, ICP8 and gC viral protein levels by Western blot. A GAPDH blot to ensure equal loading is also shown. The ratio of viral proteins to GAPDH of a representative experiment, obtained by densitometry analysis, is shown below the blots. (**B**) Immunofluorescence images of gC viral protein. Cell nuclei were stained with DAPI. Scale bar: 10 µm. (**C**) Viral DNA levels were determined by qPCR. The graph shows the data of a representative experiment (mean + standard deviation (SD) of technical replicates). (**D**) Extracellular viral titers were determined by plaque assays. The graph shows the data of a representative experiment (PFU: plaque-forming units).

To evaluate HSV-1 permissiveness in LUHMES cells, we first analyzed infection dynamics in proliferative cultures. In this undifferentiated state, LUHMES cells displayed a rounded morphology and exhibited a rapid lytic response upon HSV-1 exposure, characterized by progressive cell detachment and cytopathic effects visible at 18 h post-infection (hpi) (Supplementary Figure S1A). Western blot analysis confirmed the expression of HSV-1 proteins representative of distinct kinetic classes, including the immediate early protein ICP4, the early protein ICP8, and the true-late glycoprotein gC, all of which increased in a viral dose-dependent manner (Figure 2A). To assess the extent of infection, immunofluorescence analysis using an anti-gC antibody was performed, revealing a dose-dependent increase in the proportion of infected cells. At a multiplicity of infection (MOI) of 3 for 18 h, nearly all cells (>90%) displayed strong gC immunoreactivity (Figure 2B). qPCR further demonstrated a dose-dependent increase in HSV-1 DNA copy number (Figure 2C), consistent with active viral genome replication. Finally, viral titer assays confirmed the release of infectious viral particles into the culture supernatants, indicating that LUHMES progenitor cells efficiently support productive lytic replication of HSV-1 (Figure 2D).

**Figure 3:**
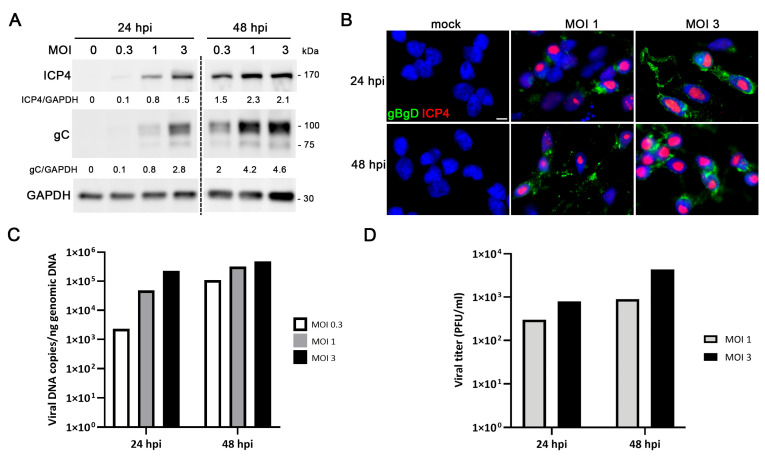
Characterization of HSV-1 infection in differentiated LUHMES neurons in 2D cultures. (**A**–**D**) LUHMES neurons differentiated for 7 days were infected with HSV-1 at different MOI for 24 and 48 h, and the infection efficiency was evaluated. (**A**) Western blot analysis of ICP4 and gC levels. A GAPDH blot to ensure equal loading is also shown. The ratio of viral proteins to GAPDH of a representative experiment is shown below the blots. (**B**) Immunofluorescence analysis using antibodies that recognize ICP4 and glycoproteins B and D. Cell nuclei were stained with DAPI. Scale bar: 10 µm. (**C**) Quantification of viral DNA levels using qPCR. The graph shows the data of a representative experiment. (**D**) Extracellular viral titers were quantified by plaque assays. The graph shows the data of a representative experiment.

To characterize HSV-1 infection in LUHMES neurons, cultures were differentiated for 7 days prior to viral exposure. At 24 hpi, neurons began to exhibit cytopathic effects, including initial signs of neurite fragmentation. By 48 hpi, neurons had lost their characteristic branched morphology, appeared rounded, and started to detach from the culture surface (Supplementary Figure S1B). Western blot analysis demonstrated the expression of HSV-1 proteins corresponding to distinct kinetic phases—ICP4 (immediate-early) and gC (late)—in differentiated neurons at 24 hpi and 48 hpi, showing a clear dependence on viral dose and infection time (Figure 3A). Consistently, immunofluorescence revealed nuclear localization of ICP4 and prominent staining of viral glycoproteins gB and gD at 24 and 48 hpi. At MOI 1, about 50% of cells expressed viral proteins, increasing to over 90% at MOI 3 (Figure 3B). qPCR confirmed active viral genome replication (Figure 3C), and titer assays detected the release of infectious particles into the culture supernatant (Figure 3D). Notably, viral yields were lower, and the appearance of extracellular virus was delayed compared with proliferative LUHMES cultures.

Together, these findings demonstrate that LUHMES cells are permissive to HSV-1 infection in both proliferative and post-mitotic neuronal states. In addition, we selected 18 hpi for proliferative LUHMES cells and 24 hpi for differentiated neurons, as these time points yielded robust and reproducible detection of viral DNA and proteins while still preserving sufficient cellular integrity for quantitative analyses. Likewise, a MOI of 3 was chosen because it provided homogeneous infection in both proliferative and differentiated cultures without inducing excessive cytotoxicity. Overall, these parameters were identified as the optimal conditions for reliably assessing HSV-1–induced AD-related molecular alterations (Aβ accumulation, tau phosphorylation and autophagy–lysosome dysfunction) in the LUHMES model.

### 2.3. HSV-1 Induces AD-Related Molecular Markers in LUHMES Cells

**Figure 4:**
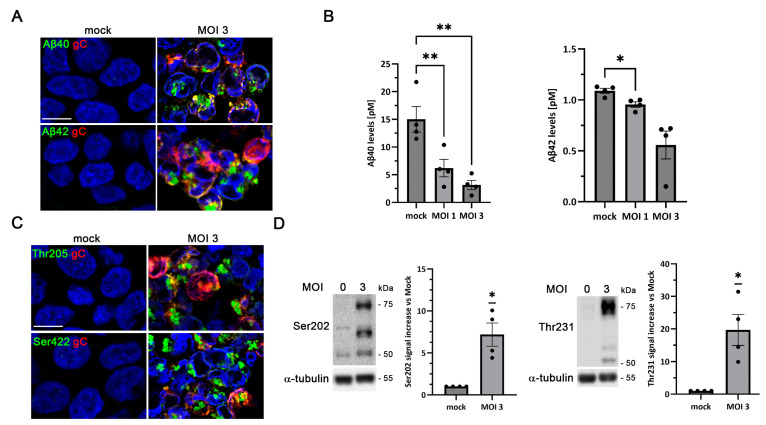
HSV-1 infection induces Alzheimer’s disease (AD)-like markers in proliferative LUHMES cells. (**A**–**D**) Proliferative LUHMES cells were infected with HSV-1 at MOI 1 or 3 for 18 h, and the beta-amyloid (Aβ) and phosphorylated tau levels were assessed. (**A**) Immunofluorescence analysis of Aβ40 and Aβ42 in HSV-1–infected and control cultures. Infection was monitored by gC staining. DAPI-stained nuclei are also shown. Scale bar: 10 µm. (**B**) Quantification of extracellular Aβ40 and Aβ42 by ELISA. The graph data represent the mean ± SEM of 4 independent experiments (*n* = 4; One-way ANOVA and Dunnett’s test; * *p* < 0.05; ** *p* < 0.01). (**C**) Immunostaining of phosphorylated tau using the phosphorylation-sensitive antibodies Thr205 and Ser422. Infection was monitored by gC staining. DAPI-stained nuclei are also shown. Scale bar: 10 µm. (**D**) Western blot analysis of phosphorylated tau (Ser202 and Thr231) levels. α-Tubulin was used as a loading control. The graph data represent the mean ± SEM of ratios of phosphorylated tau to α-tubulin obtained by densitometry analysis (*n* = 4; one-sample *t*-test; * *p* < 0.05).

Accumulation of Aβ peptides, which aggregate to form senile plaques, represents a major hallmark of AD. HSV-1 infection has been shown to promote intracellular Aβ accumulation by disrupting the non-amyloidogenic amyloid precursor protein (APP) processing pathway and reducing the secretion of Aβ peptides [16]. LUHMES cells have been reported to express endogenous APP [33,34]. We confirmed APP expression in LUHMES cells by Western blot analysis (Supplementary Figure S2A). To evaluate whether HSV-1 infection modulates Aβ levels in proliferative LUHMES cells, immunofluorescence analysis was performed. In uninfected cultures (mock), no intracellular Aβ was detected with any of the anti-Aβ antibodies tested. In contrast, following HSV-1 exposure, a pronounced accumulation of Aβ was observed, as revealed by immunolabeling with Aβ40- and Aβ42-specific antibodies (Figure 4A). The extracellular Aβ content in the conditioned media of LUHMES cells was subsequently quantified by ELISA assays. A viral dose-dependent reduction in secreted Aβ40 and Aβ42 levels was observed in HSV-1-infected cultures relative to mock-infected cultures (Figure 4B). In mock-infected cultures, Aβ40 and Aβ42 peptides are efficiently secreted, which may explain why their intracellular levels remain below the detection threshold of our immunofluorescence settings. Overall, these findings suggest that HSV-1 infection impairs Aβ secretion, thereby promoting intracellular Aβ accumulation in proliferative LUHMES cells.

Tau, a microtubule-associated protein, undergoes hyperphosphorylation in AD, leading to the formation of NFTs, another defining neuropathological feature of the disease. To assess whether HSV-1 infection affects tau phosphorylation in proliferative LUHMES cells, immunofluorescence assays were carried out using antibodies recognizing tau phosphorylated at residues Thr205 and Ser422, both of which are typically associated with NFTs. In non-infected cells, no phosphorylated tau was detected. Upon HSV-1 infection, strong accumulation of phosphorylated tau was evident at 18 hpi (Figure 4C). Western blot analysis further confirmed a significant increase in tau phosphorylation at epitopes Ser202 and Thr231, which are also present in NFTs (Figure 4D).

**Figure 5:**
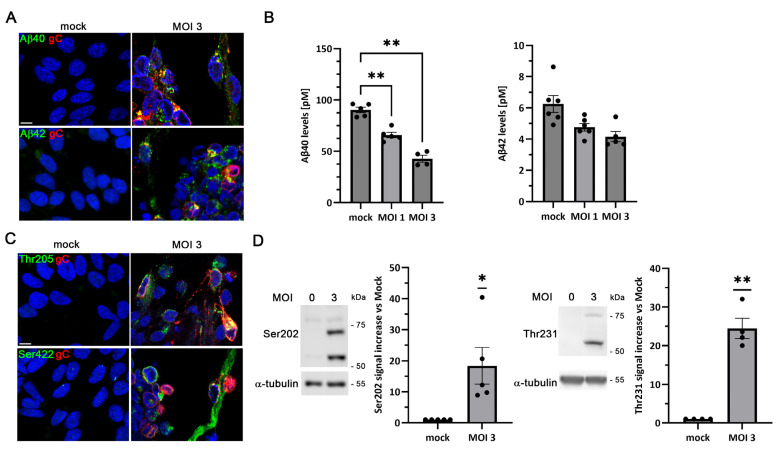
HSV-1 infection induces AD-like markers in LUHMES neurons. (**A**–**D**) 7-day-differentiated LUHMES cells were infected with HSV-1 at MOI 1 or 3 for 24 h, and the Aβ and phosphorylated tau levels were monitored. (**A**) Immunofluorescence staining of Aβ40 and Aβ42. Infection was monitored by gC staining. DAPI-stained nuclei are also shown. Scale bar: 10 µm. (**B**) Quantitative analysis of secreted Aβ40 and Aβ42 levels in conditioned medium by ELISA assays. The graph data represent the mean ± SEM of 5 (Aβ40) or 6 (Aβ42) independent experiments (*n* = 5 or *n* = 6; Mixed-effects model and Dunnett’s test; ** *p* < 0.01). (**C**) Tau phosphorylated levels were assessed by immunofluorescence using the phosphorylation-sensitive antibodies Thr205 and Ser422. Infected cells were stained with a gC antibody. DAPI-stained nuclei are also shown. Scale bar: 10 µm. (**D**) Analysis of phosphorylated tau levels by Western blot using antibodies specific for the phosphorylated epitopes Ser202 and Thr231. α-Tubulin was used as a loading control. The graph data represent the mean ± SEM of ratios of phosphorylated tau to α-tubulin obtained by densitometry analysis (*n* = 5; one-sample *t*-test; * *p* < 0.05, ** *p* < 0.01).

To determine whether the effects of HSV-1 observed in proliferative LUHMES cells were also present in mature neuronal cultures, LUHMES cells were differentiated to obtain post-mitotic neurons and subsequently infected with HSV-1. Similar to what was observed in proliferative LUHMES cells, HSV-1 infection induced a marked intracellular accumulation of Aβ in differentiated neurons. In mock-infected cultures, Aβ immunoreactivity was virtually absent, whereas HSV-1–infected neurons displayed a strong intracellular Aβ signal detected with antibodies specific to both Aβ40 and Aβ42 (Figure 5A). Consistent with these observations, ELISA quantification of conditioned media revealed a significant, dose-dependent reduction in secreted Aβ40 levels in HSV-1–infected neuronal cultures compared with non-infected controls. Although the decrease in secreted Aβ42 levels did not reach statistical significance, a clear downward trend was observed (Figure 5B). These results indicate that viral infection compromises Aβ secretion, leading to its intracellular retention in LUHMES neurons.

We next examined whether HSV-1 infection alters tau phosphorylation in these neuronal cultures. Immunofluorescence analysis revealed that, in control neurons, phosphorylated tau (Thr205 and Ser422) was barely detectable. In contrast, infected neurons exhibited widespread phospho-tau staining throughout the soma and neuritic processes at 24 hpi (Figure 5C). Western blot analysis further confirmed a significant increase in tau phosphorylation at the Ser202 and Thr231 epitopes in HSV-1-infected neuronal cultures (Figure 5D). In contrast to the increase in phospho-tau levels, HSV-1 infection induced a reduction in total tau protein levels in both undifferentiated cells and differentiated LUHMES neurons (Supplementary Figure S2B). This decrease is consistent with the global host protein shutoff associated with lytic HSV-1 infection and indicates that the increase in phospho-tau signal is not merely a consequence of elevated total tau levels, but rather reflects a genuine increase in tau phosphorylation. Finally, to exclude the possibility that the detected phospho-tau signals corresponded to viral proteins, we performed a control Western blot in which only the viral preparation was loaded (Supplementary Figure S2C). Under these conditions, no bands corresponding to phospho-tau epitopes (Ser202 or Thr231) were detected, confirming the specificity of the antibodies used.

Collectively, these results demonstrate that HSV-1 infection recapitulates hallmark AD-like pathological features—intracellular Aβ accumulation and tau hyperphosphorylation—not only in proliferative LUHMES cells but also in differentiated LUHMES-derived neurons. These findings underscore the capacity of HSV-1 to alter the metabolism of amyloid peptides and the phosphorylation of tau protein across different stages of neuronal maturation, thereby strengthening the potential of this human neuronal model for investigating the pathogenic link between viral infection and AD-related alterations.

### 2.4. HSV-1 Alters Markers of the Autophagy–Lysosome Pathway in LUHMES Cells

**Figure 6:**
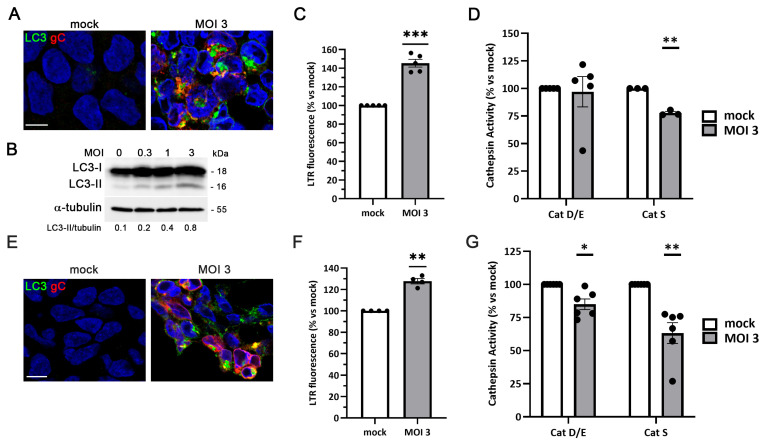
Lysosomal alterations induced by HSV-1 in proliferative and differentiated LUHMES cells. (**A**–**D**) LUHMES proliferative cells were infected with HSV-1 at different MOI for 18 h. (**A**) Immunofluorescence images of intracellular LC3 accumulation in infected cells (MOI 3) are shown. Infection was monitored by gC staining. DAPI-stained nuclei are also shown. Scale bar: 10 µm. (**B**) Western blot analysis of LC3 in LUHMES cell lysates after HSV-1 infection at different viral doses. An α-tubulin blot to ensure equal loading is also shown. The ratio of LC3-II to tubulin of a representative experiment, obtained by densitometric analysis, is shown below the blots. (**C**) Analysis of lysosomal load by quantification of the LysoTracker Red (LTR) fluorescence in LUHMES cells infected with HSV-1 at MOI 3. The graph data show the mean ± SEM of 5 independent experiments (*n* = 5; one-sample *t*-test; *** *p* < 0.001). (**D**) The relative enzymatic activities of cathepsins D/E and S were quantified in LUHMES cells after HSV-1 infection (MOI 3). Graph data show the mean ± SEM of 3 (Cat S) or 5 (Cat D/E) independent experiments (*n* = 3 or *n* = 5; one-sample *t*-test; ** *p* < 0.01). (**E**–**G**) LUHMES neurons, differentiated for 7 days, were infected with HSV-1 at an MOI of 3 for 24 h, and lysosomal alterations were analyzed. (**E**) Immunofluorescence images of intracellular LC3. Infection was monitored with an antibody specific for gC. Cell nuclei were stained with DAPI. Scale bar: 10 µm. (**F**) Lysosomal load was determined by quantifying LTR fluorescence. The graph data show the mean ± SEM of 4 independent experiments (*n* = 4; one-sample *t*-test; ** *p* < 0.01). (**G**) Lysosomal activity of cathepsins D/E and S was measured. The graph data show the mean ± SEM of 6 independent experiments (*n* = 6; one-sample *t*-test; * *p* < 0.05, ** *p* < 0.01).

Another neuropathological mechanism associated with AD and reported to be affected by HSV-1 infection involves the dysfunction of the autophagy–lysosome pathway. Among numerous lines of evidence supporting this relationship, we have reported alterations in this axis in human neuroblastoma cells [35]. During autophagy activation, the cytosolic form of LC3 (LC3-I) undergoes lipidation to form LC3-II, which binds to autophagic membranes and remains associated with autophagosomes throughout their maturation. To determine whether HSV-1 alters this pathway in LUHMES cells, we first analyzed LC3 expression levels and distribution in proliferative cultures. Immunofluorescence analysis revealed a marked accumulation of LC3 in HSV-1-infected cells, whereas LC3 staining was nearly undetectable in mock-infected cultures (Figure 6A). The punctate LC3 staining pattern was consistent with the accumulation of autophagosomes. Western blot analysis corroborated these observations, showing a viral dose-dependent increase in LC3-II levels (Figure 6B).

To further explore the effects of HSV-1 on lysosomal integrity and function, we evaluated the lysosomal load using the lysosomotropic dye LysoTracker Red (LTR), as well as the enzymatic activity of several lysosomal hydrolases (cathepsins D/E and S). LTR fluorescence intensity was significantly higher in HSV-1–infected LUHMES cultures than in non-infected controls, reflecting an increased lysosomal burden upon infection (Figure 6C). Moreover, while cathepsin D/E activity was not affected by infection, the activity of lysosomal cathepsin S was significantly reduced in HSV-1–infected LUHMES cells (Figure 6D), suggesting a defect in lysosomal proteolytic function. Together, these data indicate that HSV-1 disrupts the autophagy–lysosome pathway in proliferative LUHMES cells, suggesting altered autophagic processing and lysosomal degradation, although additional flux-specific assays would be required to definitively distinguish enhanced autophagy induction from impaired clearance.

We next investigated whether similar alterations occurred in LUHMES cells differentiated into mature neurons. Immunofluorescence analysis revealed an accumulation of LC3 in HSV-1-infected neurons (Figure 6E), consistent with an accumulation of autophagosomes as observed in proliferative LUHMES cells. In contrast to mock-infected controls, differentiated neurons exposed to HSV-1 exhibited a pronounced increase in LTR fluorescence intensity (Figure 6F), indicating that viral infection also enhances lysosomal content in mature neuronal cultures. Furthermore, lysosomal enzymatic activity measured using fluorogenic substrates for cathepsins D, E, and S showed a significant decrease in all tested enzymes in infected neuronal cultures (Figure 6G). These results indicate that HSV-1 infection compromises both the autophagic process and lysosomal proteolytic activity in LUHMES-derived neurons.

Together, these findings suggest that HSV-1-induced alterations of the autophagy and lysosome machineries may represent a relevant mechanism underlying the appearance of AD-related markers in the infected neurons.

### 2.5. Development of a 3D Neuronal Model Using Differentiated LUHMES Cells

**Figure 7:**
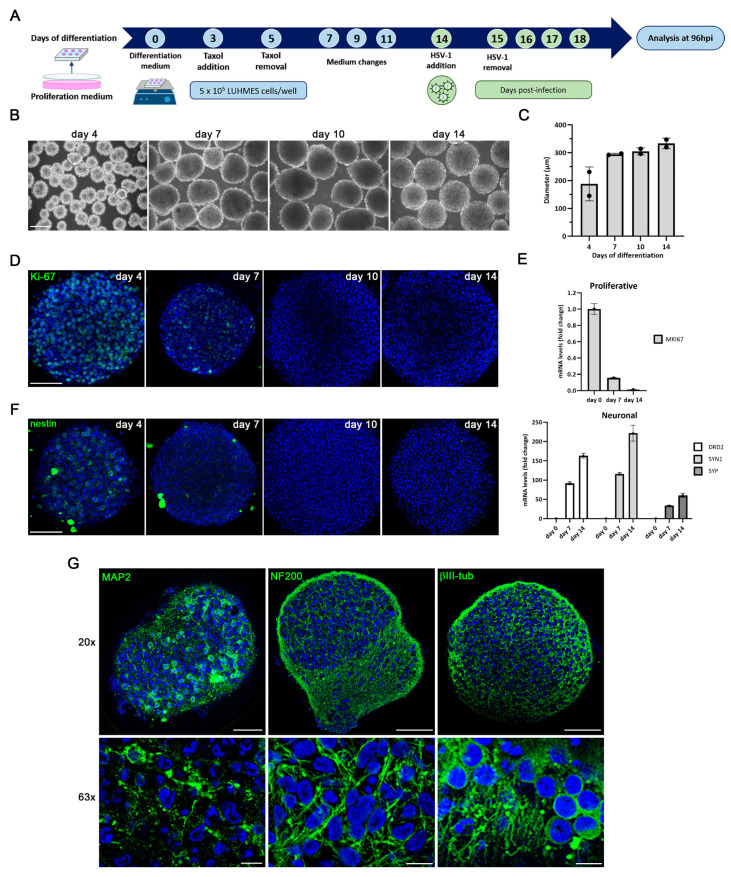
Characterization of three-dimensional (3D) LUHMES neuronal cultures. (**A**) The two-dimensional (2D) differentiation protocol was adapted for 3D culture by maintaining the single-cell suspension under continuous gyratory shaking. The infection with HSV-1-UL46-GFP was added at day 14 of differentiation, and 3D aggregates were processed at 96 hpi. (**B**) Phase-contrast microscopy images of 3D LUHMES neuronal cultures at different days of differentiation. Scale bar: 200 µm. (**C**) Quantification of the diameter of 3D aggregates at days 4, 7, 10 and 14 of differentiation. The graph shows the mean ± SD of the diameter of at least 50 3D aggregates per condition. (**D**) Immunofluorescence analysis of 3D neuronal cultures showing Ki-67–positive cells at differentiation days 4, 7, 10, and 14. DAPI-stained nuclei are also shown. Scale bar: 100 μm. (**E**) Analysis of gene expression of progenitor (*MKI67*) and neuronal (*DRD2, SYN1* and *SYP*) genes by RT-qPCR in undifferentiated (day 0) and 7 and 14-day LUHMES differentiated cells. Graph data show the mean ± SD of a representative experiment. (**F**) Immunofluorescence images of 3D LUHMES cultures at differentiation days 4, 7, 10 and 14, stained with an antibody against the neural progenitor cell marker nestin. Scale bar: 100 μm. (**G**) Immunofluorescence images showing neuronal markers (MAP2, neurofilament heavy chain (NF200), and βIII-tub) in 14-day differentiated 3D LUHMES cultures. Higher-magnification micrographs are also shown. Cell nuclei were stained with DAPI. Scale bars: 100 μm (20×) and 10 μm (63×).

Building on these findings, we next sought to determine whether HSV-1 infection induces similar AD-like alterations within a more structured 3D neuronal environment. To this end, we first generated and optimized LUHMES-based 3D neuronal cultures. Following a previously described protocol for the establishment of 3D LUHMES neuronal cultures [26], we adapted and refined the procedure to fit our experimental conditions (Figure 7A). Briefly, LUHMES cells were seeded in low-adhesion plates containing differentiation medium and maintained under constant orbital agitation. Under these conditions, the cells spontaneously aggregated into free-floating spherical structures (Figure 7B). To ensure appropriate oxygen and nutrient diffusion and to prevent the formation of necrotic cores, the aggregate diameter was kept below 350 µm. Continuous monitoring of cultures up to day 14 of differentiation revealed stable aggregate morphology, with an average diameter of approximately 300 µm (Figure 7C).

To assess the proliferation rate within 3D LUHMES cultures, we analyzed the expression of Ki-67, a well-established proliferation marker, in undifferentiated cells and at 4, 7, 10, and 14 days of differentiation. Immunofluorescence analysis revealed a clear Ki-67 signal at day 4, with Ki-67–positive cells distributed throughout the aggregates, indicating the presence of actively dividing cells. As differentiation progressed, the number of Ki-67-positive cells markedly decreased and became nearly undetectable by day 10 (Figure 7D), consistent with a progressive inhibition of proliferation during neuronal maturation.

To further characterize the differentiation status of the 3D LUHMES cultures, RT-qPCR analysis was performed to quantify the expression of neuronal and progenitor markers throughout the differentiation process. The expression levels of the proliferation marker gene *MKI67* and the neuronal genes *DRD2*, *SYN1*, and *SYP* were analyzed relative to those in undifferentiated cells. Consistent with the immunofluorescence data, Ki-67 mRNA levels progressively declined during differentiation, indicating the loss of proliferative and progenitor features. In contrast, neuronal markers were upregulated, reflecting the acquisition of a mature neuronal phenotype. By day 14 of differentiation, expression of neuronal genes reached maximal levels, while *MKI67* expression was strongly downregulated (Figure 7E).

Consistent with these transcriptional data, immunofluorescence analysis further confirmed neuronal differentiation within 3D aggregates. Staining for the neural progenitor marker nestin revealed a marked reduction in its expression as differentiation progressed, indicating the loss of precursor characteristics (Figure 7F). In contrast, mature neuronal markers such as MAP2, neurofilament heavy chain (NF200), and βIII-tubulin showed strong and widespread expression throughout the aggregates, consistent with a well-established neuronal phenotype. High-magnification images highlighted extensive neuritic arborization and the presence of long, branching neurites characteristic of mature neurons, confirming the proper neuronal maturation achieved in the 3D cultures (Figure 7G).

These findings demonstrate that by day 14, LUHMES cells within 3D aggregates acquire a mature neuronal phenotype characterized by increased neuronal gene expression, extensive neurite formation, and an almost complete loss of proliferative markers.

### 2.6. Development of a 3D LUHMES-Based Model of HSV-1 Infection

**Figure 8:**
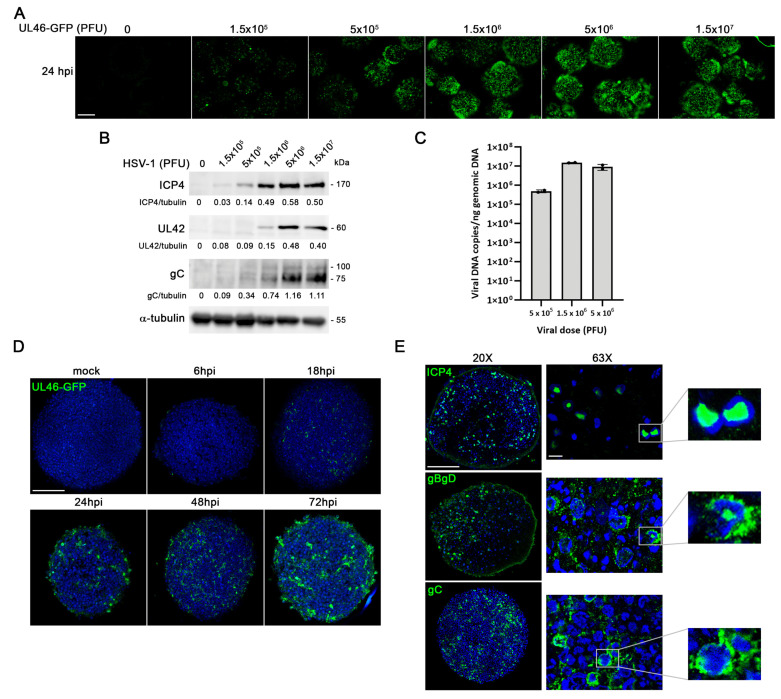
Analysis of HSV-1 infection in LUHMES neuronal aggregates. (**A**) Fluorescence microscopy images of LUHMES 3D cultures infected for 24 h with increasing doses of the fluorescent HSV-1 strain UL46-GFP. Scale bar: 200 μm. (**B**,**C**) LUHMES 3D neuronal cultures differentiated for 14 days were infected for 96 h with increasing doses of UL46-GFP strain. (**B**) Analysis of ICP4, UL42, and gC viral protein levels by Western blot. An α-tubulin blot to ensure equal loading is also shown. The ratio of viral proteins to α-tubulin of a representative experiment is shown below the blots. (**C**) Viral DNA levels were quantified by qPCR. Data represent the mean ± SD of a representative experiment. (**D**) 14 day-differentiated 3D LUHMES cultures were infected with 5 × 10^6^ PFU of UL46-GFP strain for different time periods. Confocal microscopy images corresponding to the center of the 3D aggregates illustrate the time-dependent penetration of HSV-1 infection. Cell nuclei were stained with DAPI. Scale bar: 100 μm. (**E**) Immunofluorescence analysis using antibodies against ICP4 and viral glycoproteins B, D and C in 14-day-differentiated 3D LUHMES cultures infected with 5 × 10^6^ PFU for 96 h. Higher-magnification images show the nuclear localization of ICP4 and the cytosolic distribution of viral glycoproteins. Cell nuclei were stained with DAPI. Scale bars: 100 µm (20×) and 10 µm (63×). In all panels, spheroids were optically cleared and imaged using confocal z-stacks. Panels show central optical sections.

One of the main objectives of this study was to establish a model of HSV-1 infection in 3D LUHMES neuronal cultures. To optimize infection conditions, we used a fluorescent HSV-1 strain expressing the tegument protein VP11/12—encoded by the UL46 gene—as a GFP fusion protein (UL46-GFP). 3D LUHMES cultures were initially infected with increasing doses of the UL46-GFP strain for 24 h, and infection efficiency was assessed by fluorescence microscopy. A dose-dependent increase in both the extent and intensity of GFP fluorescence within the 3D aggregates was observed, indicating the progressive spread of infection throughout the cultures (Figure 8A).

Subsequently, the expression of viral proteins representative of different stages of the viral replication cycle was quantified: ICP4 (an immediate-early gene), UL42 (a leaky late gene), and gC (a true late gene). Western blot analysis showed a dose-dependent increase in all three viral proteins, reaching a plateau at a viral dose of 5 × 10^6^ plaque-forming units (PFU) (Figure 8B). To further confirm active viral replication, viral DNA levels were quantified by qPCR, demonstrating that the UL46-GFP strain replicates efficiently in 3D LUHMES neuronal cultures (Figure 8C). Taken together, these results confirm the susceptibility of 3D LUHMES cultures to HSV-1 infection and allowed the optimization of experimental parameters, establishing 5 × 10^6^ PFU as the optimal viral dose for subsequent assays.

To further assess the ability of the virus to penetrate the 3D aggregates, confocal optical sections corresponding to the central regions of the spheroids were analyzed at different post-infection time points. Confocal microscopy images revealed that the viral signal became detectable in the inner layers of the aggregates at 18 hpi and progressively increased over time (Figure 8D). These observations confirm that HSV-1 efficiently spreads throughout the entire 3D structure, resulting in a homogeneous infection across the aggregates.

Finally, immunofluorescence assays were performed to confirm the expression and localization of viral proteins within 3D LUHMES aggregates after 96 h of infection. Confocal microscopy images showed the presence of ICP4 and the glycoproteins gB, gC and gD distributed throughout the aggregates. High-magnification images revealed the characteristic nuclear localization of ICP4 within viral replication compartments, while gB, gC, and gD were detected predominantly in the cytoplasm (Figure 8E). These observations confirm that HSV-1 undergoes a complete and efficient infection in 3D LUHMES neuronal cultures.

Altogether, we determined that infection of 3D LUHMES aggregates with 5 × 10^6^ PFU and analysis at 96 hpi provided the most consistent and homogeneous viral infection while preserving sufficient aggregate integrity to evaluate HSV-1–induced neurodegeneration-related markers. These parameters were therefore selected as the optimal conditions for the 3D model.

### 2.7. HSV-1 Triggers AD-like Alterations in 3D LUHMES Neuronal Cultures

Our next aim was to determine whether the pathological effects of HSV-1 observed in proliferative and differentiated 2D LUHMES cultures were recapitulated in 3D neuronal aggregates. To this end, we analyzed the accumulation and secretion of Aβ peptides, as well as the phosphorylation state of tau.

**Figure 9:**
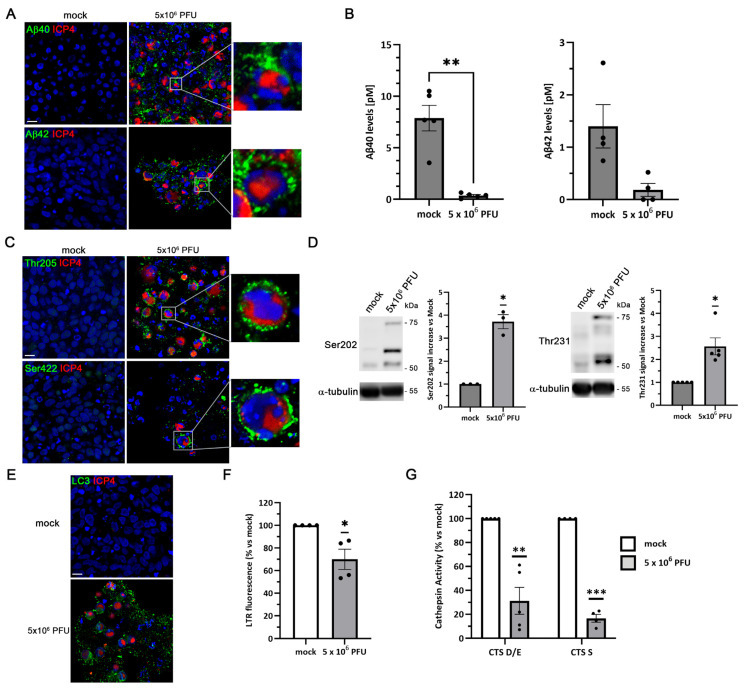
HSV-1 infection triggers Aβ accumulation, tau hyperphosphorylation, and lysosomal dysfunction in 3D LUHMES neuronal cultures. LUHMES 3D neuronal cultures were infected with 5 × 10^6^ PFU of HSV-1 for 96 h, and Aβ levels, tau phosphorylation, and lysosomal alterations were assessed. (**A**) Immunofluorescence analysis of Aβ40 and Aβ42 accumulation in HSV-1–infected and control cultures. Infection was monitored by ICP4 staining. Higher-magnification images show Aβ staining in infected cells. Nuclei were counterstained with DAPI. Scale bar: 10 µm. (**B**) Quantification of extracellular Aβ40 and Aβ42 levels by ELISA. Data represent the mean ± SEM of 4 (Aβ42) or 5 (Aβ40) independent experiments (*n* = 4 or *n* = 5; Student’s *t*-test; ** *p* < 0.01). (**C**) Immunostaining of phosphorylated tau using phosphorylation-sensitive antibodies Thr205 and Ser422. Infection was monitored by ICP4 staining. Higher-magnification images show phosphorylated tau staining in infected cells. Nuclei were counterstained with DAPI. Scale bar: 10 µm. (**D**) Western blot analysis of phosphorylated tau (Ser202 and Thr231) levels. An α-tubulin blot is shown as a loading control. The graph data represent the mean ± SEM of ratios of phosphorylated tau to α-tubulin obtained by densitometry analysis (*n* = 3 for Ser202 and *n* = 5 for Thr231; one-sample *t*-test; * *p* < 0.05). (**E**) Immunofluorescence images of intracellular LC3 accumulation. Infection was monitored with an antibody specific for ICP4. Cell nuclei were stained with DAPI. Scale bar: 10 µm. (**F**) Lysosomal load was determined by quantifying LTR fluorescence. The graph data show the mean ± SEM of 4 independent experiments (*n* = 4; one-sample *t*-test; * *p* < 0.05). (**G**) Enzymatic activity of cathepsins D/E and S was measured. The graph data show the mean ± SEM of 4 (Cat S) or 5 (Cat D/E) independent experiments (*n* = 4 or *n* = 5; one-sample *t*-test; ** *p* < 0.01; *** *p* < 0.001). In all immunofluorescence panels, spheroids were optically cleared and imaged using confocal z-stacks. Panels show central optical sections.

In non-infected 3D cultures, no detectable signal for Aβ or phosphorylated tau was observed. In contrast, HSV-1-infected cultures exhibited clear intracellular Aβ accumulation, as revealed by immunofluorescence using antibodies specific for Aβ40 and Aβ42. Higher magnification images showed that Aβ accumulation occurred in ICP4-positive cells, indicating its association with active viral infection (Figure 9A). ELISA quantification of extracellular Aβ confirmed a strong reduction in secreted Aβ40 and Aβ42 levels, which was statistically significant in the case of Aβ40 (Figure 9B). Furthermore, immunostaining with phospho-tau-specific antibodies revealed a robust accumulation of phosphorylated tau at Thr205 and Ser422 epitopes in infected neurons, forming a punctate cytoplasmic pattern similar to that previously described in 2D LUHMES cultures (Figure 9C). Consistently, Western blot analysis revealed a significant increase in tau phosphorylation at the Ser202 and Thr231 epitopes in HSV-1–infected 3D neuronal cultures (Figure 9D), further supporting the induction of AD-like pathological features by the virus.

To investigate whether the lysosomal dysfunction previously detected in 2D LUHMES neurons also occurs under 3D conditions, we examined markers of the autophagy–lysosome pathway in infected aggregates. Immunofluorescence analysis showed a pronounced accumulation of LC3 in infected neurons, suggesting altered autophagosome dynamics (Figure 9E). In parallel, a significant decrease in LTR fluorescence was detected, suggesting a loss of lysosomal acidity and/or reduced lysosomal quantity (Figure 9F). Consistent with these observations, the enzymatic activity of lysosomal cathepsins D, E and S was markedly decreased in infected 3D cultures compared with controls, indicating impaired proteolytic capacity (Figure 9G). Collectively, these findings demonstrate that HSV-1 infection disrupts lysosomal function in differentiated 3D LUHMES neurons, mirroring the alterations previously identified in 2D models and supporting a conserved mechanism of virus-induced lysosomal dysfunction.

In summary, the 3D LUHMES neuronal model faithfully recapitulates the AD-like neuropathological alterations induced by HSV-1, providing a useful human neuronal system to study HSV-1–induced AD-related molecular mechanisms. These data further strengthen the hypothesis that HSV-1 may act as an environmental factor contributing to AD pathogenesis by disrupting neuronal homeostasis and protein clearance mechanisms.

## 3. Discussion

AD is the most common cause of dementia and represents an increasing global health challenge. Despite extensive research, the precise mechanisms underlying AD pathogenesis remain incompletely understood, and effective disease-modifying therapies are still lacking. The growing prevalence of AD highlights the urgent need for experimental models that more faithfully reproduce the complexity of the human disease and overcome the limitations of current research systems. The present study provides significant insights into the potential link between HSV-1 infection and AD-related molecular alterations, specifically emphasizing the value of LUHMES-derived neuronal models in understanding this relationship. Our results reinforce the growing body of evidence positioning HSV-1 as a meaningful environmental risk factor contributing to AD pathogenesis.

The LUHMES cell line, derived from human embryonic mesencephalic precursor cells, offers critical advantages for studying infection-associated neurodegeneration. These cells exhibit both proliferative and neuronal differentiation states [24], enabling the examination of infection from initial viral entry to mature neuronal dysfunction. Their stable and homogeneous differentiation into neuron-like cells under defined conditions provides high reproducibility—an essential feature for mechanistic dissection and pharmacological screening.

Despite dopaminergic neurons have been extensively studied in the context of other neurodegenerative diseases, such as Parkinson’s disease, alterations in dopaminergic midbrain have recently gained interest for AD research [36]. Moreover, the trigeminal ganglia—one of the entry paths to the CNS proposed for HSV-1—is also connected to the brainstem, suggesting a plausible way for the virus to access and impair the dopaminergic system. In fact, a recent study investigated the spatial pattern of HSV-1 infection in mice, and reported the presence of viral antigens in different brain regions, including brainstem’s neuromodulatory centers [37]. Thus, dopaminergic nature of LUHMES neurons seems also adequate to assess the role of HSV-1 in AD.

Another major strength of the LUHMES model is its adaptability to both 2D and 3D culture systems [26]. While 2D cultures facilitate high-throughput exploration of viral and host responses, 3D cultures more closely reproduce the spatial organization, cell–cell communication, and cell-matrix interactions that take place in the human brain. Nevertheless, these platforms still lack other components that are essential for modelling brain pathophysiology, such as glial cells, which are involved in neuroinflammation. In this regard, we are currently working with neuro-glial models of HSV-1 infection and neurodegeneration [38], which could provide a complementary view to the tools presented in this work.

The LUHMES neuronal model also provides an effective human platform for studying how neurotropic viruses disrupt neuronal homeostasis. Previous studies have demonstrated that LUHMES neurons can be efficiently infected by HSV-1, supporting both lytic and latent infection modes in 2D systems [31,39]. Beyond HSV-1, LUHMES progenitors and differentiated neurons are also permissive to other neurotropic viruses, including hemorrhagic fever and Zika viruses [30], further validating the model’s versatility for studying virus–neuron interactions.

One of the main goals of this study was to establish a model of lytic HSV-1 infection in 3D LUHMES neuronal cultures. Using an orbital shaking-based method originally developed for human induced pluripotent stem cells (iPSCs)-derived neurons [26,40], we generated uniform spheroidal aggregates that mimic key features of neuronal architecture. The results obtained from the evaluation of various viral parameters demonstrated the ability of HSV-1 to efficiently infect different types of LUHMES cultures, including both 2D proliferative and differentiated cells, as well as 3D neuronal aggregates. First, a dose-dependent increase in the expression of viral proteins was observed. Since these proteins are expressed at distinct stages of the viral replication cycle (ICP4 is an α gene expressed at the immediate-early stage; gB is a β gene expressed during the early phase; and gC and gD are “true late” γ2 genes whose expression strictly depends on viral DNA replication), these findings suggest that the virus is capable of completing its full replication cycle in 3D aggregates. Second, qPCR analysis revealed the presence of HSV-1 DNA in the 3D neuronal cultures, confirming the replicative capacity of the virus. Finally, immunofluorescence assays detected the expression of ICP4 and the viral glycoproteins gC and gB/gD throughout the entire 3D aggregates. Collectively, these data demonstrate the ability of HSV-1 to efficiently infect and replicate in 3D cultures of LUHMES-derived neurons, underscoring the model’s potential for studying virus–induced neurodegeneration.

We further show that HSV-1 infection elicits hallmark AD-like alterations in proliferative and differentiated LUHMES cells across both 2D and 3D culture systems, including intracellular Aβ accumulation and robust tau hyperphosphorylation. These findings extend our previous observations in other neuronal cell systems [16,17,38,41], and strengthen the concept that HSV-1 can simultaneously disrupt Aβ metabolism and tau regulation—two central drivers of AD pathology. These observations are consistent with epidemiological studies and neuropathological analyses identifying HSV-1 DNA in brain regions significantly affected by AD [15,42]. The accumulation of Aβ peptides accompanied by a reduction in their extracellular secretion, suggests that HSV-1 interferes with the proteolytic processing of APP, potentially through the modulation of α-, β- and γ-secretase activity. The inhibition of Aβ secretion could underlie the intracellular accumulation of Aβ induced by HSV-1, as observed in other cellular models of infection [16]. Given the proposed antimicrobial function of Aβ [43], this buildup may initially reflect an innate antiviral response that, if chronically activated, could contribute to neurotoxicity and plaque formation.

In parallel, the marked increase in tau phosphorylation at multiple AD-related epitopes (Ser202, Thr205, Thr231, and Ser422) highlights the ability of HSV-1 to disturb tau homeostasis. This extensive pattern of hyperphosphorylation suggests broad dysregulation of kinase activity, and is compatible with the involvement of several tau-related kinases described in the literature (e.g., GSK3β, CDK5, p38 MAPK) [44]. However, because kinase activation was not directly assessed in this study, the contribution of specific signaling pathways remains hypothetical and will require further investigation. Notably, our group previously demonstrated that inhibitors of several CDKs were able to reverse tau phosphorylation at Ser396, Ser404, and Ser409 epitopes in an HSV-1 infection model using the human neuroblastoma cell line SK-N-MC [17], further supporting the involvement of CDK-related pathways in HSV-1–induced tau dysregulation. Similar phosphorylation profiles in both proliferative and differentiated LUHMES cells indicate that this disruption is independent of maturation stage, reflecting a generalized perturbation of tau regulation by HSV-1.

Beyond classical neuropathological markers, our results reveal HSV-1-induced impairment of autophagy–lysosome function, evidenced by LC3-II accumulation, impaired lysosomal burden, and reduced cathepsin activity. These data are consistent with a possible alteration in autophagosome maturation and/or lysosomal acidification/enzymatic function, although LC3-II accumulation alone cannot distinguish impaired clearance from enhanced initiation of autophagy. Because autophagic flux was not directly measured in this study, interpretations regarding impaired autophagosome clearance must be made with caution. The combination of LC3-II accumulation, reduced cathepsin activity and altered lysosomal markers suggests lysosomal dysfunction, but does not by itself establish a definitive block in autophagic flux. Consistent with this, our group previously reported that HSV-1 infection in human neuroblastoma cells leads to inhibition of late stages of the autophagic process and profound dysfunction of the lysosomal pathway, reinforcing the notion that this virus profoundly interferes with cellular degradative systems [35,41]. Such alterations are known to occur early in AD and provide a mechanistic link between defective proteostasis, Aβ accumulation, and tau dysregulation [20]. Thus, HSV-1 may not only mimic but also accelerate the neurodegenerative cascade through sustained impairment of cellular clearance pathways.

Taken together, our findings demonstrate that HSV-1 can reproduce core AD-like pathological signatures across different stages of neuronal differentiation. The ability of the virus to modulate both APP processing and tau phosphorylation highlights its potential role as a multifactorial driver of neurodegeneration. These data support the growing body of evidence linking latent or recurrent HSV-1 infection to the molecular events underlying sporadic AD [45,46,47]. Further investigation using 3D LUHMES-based cultures and single-cell transcriptomic approaches will help elucidate the cellular pathways and signaling networks through which HSV-1 contributes to neurodegenerative processes in the human brain. It is important to note that this study is based on an acute, high-dose lytic infection paradigm, which does not replicate the chronic, latent, or intermittently reactivating HSV-1 infection scenario proposed to contribute to AD over decades. Therefore, the alterations described here should be interpreted as acute neuronal responses to lytic infection, and their long-term relevance to latent or recurrent HSV-1 infection will require further investigation.

To date, only a few studies have employed LUHMES cells to investigate HSV-1 infection, and most of these have focused on establishing and characterizing latent infection in conventional 2D cultures [31,32,39]. Consequently, the effects of productive HSV-1 infection and its impact on neuronal integrity, protein homeostasis, and AD-like pathological hallmarks have not been explored. Although several 3D models of HSV-1 infection have been previously reported in different human neuronal systems [48,49,50], to our knowledge, this work reports for the first time the establishment of a 3D LUHMES model of HSV-1 infection, which recapitulates major AD-related features in a human neuronal context. This represents a step forward in modelling HSV-1-induced neurodegenerative mechanisms in a controlled human neuronal system. A priority for future work in our group is to expand this system to model HSV-1 latency and reactivation. HSV-1 latency has been successfully established in 2D LUHMES neuronal cultures [31]. Although a few studies have described latent HSV-1 infection in 3D brain organoids derived from human iPSCs [49,51], these models often display high complexity and heterogeneity, which may limit mechanistic analyses. To our knowledge, no 3D model of latent HSV-1 infection has yet been developed using LUHMES-derived neurons, to investigate the latent state or to evaluate how reactivation contributes to neuronal dysfunction and neurodegeneration. The implementation of such a 3D latency model would represent a valuable tool for elucidating the molecular mechanisms by which HSV-1 persistence leads to progressive neuronal damage and AD-related pathology.

In conclusion, our study underscores the potential of LUHMES-derived neuronal models as a translational platform to elucidate the interplay between HSV-1 infection and AD pathology. The ability to simulate key neuropathological features of AD in response to HSV-1 infection within these human-derived neuronal cells provides a powerful framework for future research. Ultimately, insights derived from these models may significantly advance our understanding of virus-induced neurodegeneration, uncover potential therapeutic targets, and guide the development of effective interventions to mitigate HSV-1-associated neurodegenerative processes.

### Limitations of the Study

While this study establishes LUHMES cells as a robust human neuronal platform to investigate HSV-1–induced AD-related molecular alterations, several limitations should be acknowledged.

First, although LUHMES cells differentiate into homogeneous populations of post-mitotic human neurons, they are of mesencephalic dopaminergic origin and therefore do not fully recapitulate the cortical or hippocampal neuronal subtypes primarily affected in AD. Nevertheless, increasing evidence supports a role for dopaminergic midbrain alterations in disease development, and the trigeminal ganglia—one of the proposed HSV-1 entry routes into the central nervous system—are connected to the brainstem, providing a plausible pathway for viral access to dopaminergic circuits. In addition, the LUHMES model lacks other central nervous system cell types, including astrocytes, microglia, oligodendrocytes, vascular and immune cells, which are known to modulate neurodegenerative processes in vivo. Consequently, this system does not aim to reproduce full brain complexity but rather offers a reductionist neuronal framework to study virus-induced molecular alterations under controlled conditions.

Second, the experimental paradigm employed relies on acute lytic HSV-1 infection, whereas the epidemiological association between HSV-1 and Alzheimer’s disease is more plausibly linked to lifelong latent infection with intermittent reactivation. Although acute infection is a widely used and informative approach to uncover virus-driven molecular pathways, the present study does not address latency, reactivation cycles or chronic low-grade infection, which represent important directions for future research.

Third, while multiple AD-related markers were analyzed—including intracellular Aβ accumulation, tau phosphorylation and autophagy–lysosome pathway alterations—the study focuses primarily on molecular and cellular readouts. Functional neuronal outcomes, such as electrophysiological activity, synaptic integrity or network connectivity, were not assessed and therefore neurodegeneration is inferred at the molecular level rather than demonstrated through functional impairment.

Finally, although the LUHMES system offers practical advantages in terms of reproducibility, scalability and suitability for both 2D and 3D cultures, the findings presented here should be interpreted as mechanistic and exploratory, rather than directly translatable to clinical settings. Further validation in more complex human-based models—such as brain organoids, co-culture systems including glial cells, or in vivo models—will be required to assess the broader physiological and clinical relevance of HSV-1–induced alterations described in this study.

Despite these limitations, the LUHMES-based platform described here provides a valuable and accessible human neuronal model to dissect virus-induced molecular mechanisms relevant to neurodegenerative processes and to support future mechanistic and translational studies.

## 4. Materials and Methods

### 4.1. Cell Culture and Differentiation

LUHMES human neuronal precursor cells (BioCat GmbH, Heidelberg, Germany) were cultured as previously described [24]. Briefly, cells were maintained in Nunclon Delta-treated culture plates (ThermoFisher Scientific, Waltham, MA, USA) pre-coated with 50 μg/mL poly-L-ornithine (Sigma-Aldrich, St. Louis, MO, USA) and 1 μg/mL fibronectin (Sigma-Aldrich). Cells were cultured in proliferation medium composed of Advanced DMEM/F12 supplemented with GlutaMAX^TM^ (Gibco, Waltham, MA, USA), 1% N2 supplement (Gibco), 50 μg/mL gentamicin and 40 ng/mL recombinant basic fibroblast growth factor (bFGF; R&D Systems, Minneapolis, MN, USA). Cell passages were performed every 3–4 days.

Neuronal differentiation of LUHMES cultures was performed following previously established protocols [25,26]. For 2D differentiation, cells were switched to differentiation medium containing Advanced DMEM/F12 supplemented with GlutaMAX^TM^, 1% N2 supplement, 50 μg/mL gentamicin and 2 μg/mL tetracycline (Sigma-Aldrich), and maintained for 7 days with medium changes every 2–3 days. For 3D differentiations, 5 × 10^5^ LUHMES cells were seeded in 6-well non-treated plates (ThermoFisher Scientific) containing differentiation medium and cultured under orbital agitation at 90 rpm (Celltron shaker system; INFORS HT, Bottmingen, Switzerland). The medium was renewed every 2–3 days. To inhibit residual proliferation, taxol (10 nM; paclitaxel, Sigma-Aldrich) was added on day 3 of differentiation and removed two days later. 3D neuronal aggregates were maintained in differentiation medium for 14 days. After 7 days in 2D cultures and 14 days in 3D cultures, neuronal maturation was assessed by phase-contrast microscopy and by evaluating marker expression using RT-qPCR, Western blot, and immunofluorescence assays.

All LUHMES cultures were incubated at 37 °C in a humidified atmosphere containing 5% CO_2_.

### 4.2. HSV-1 Infection

2D LUHMES cultures were infected with the wild-type HSV-1 strain KOS 1.1 (kindly provided by Dr. L. Carrasco) at different MOI and for various time points as indicated in each experiment. Cells were incubated in a viral solution for 1 h at 37 °C. Then, the unbound virus was removed and replaced with fresh culture medium. Cells were maintained at 37 °C until their collection. Differentiated 2D cultures were infected after 7 days of differentiation.

3D LUHMES aggregates were infected after 14 days of differentiation using different viral doses and incubation times, as specified in each experiment. A fluorescent HSV-1 strain expressing GFP fused to the UL46 tegument protein (UL46-GFP) [52] was also used in selected assays. At 18 hpi, the culture medium was replaced to remove residual extracellular virus, and samples were collected at 4 days post-infection.

Control cultures (mock) were incubated with virus-free suspensions and processed using identical procedures. Both HSV-1 strains were propagated and purified from Vero cells, as previously described [53]. Viral titers in cell culture supernatants were determined by plaque assays as previously described [54].

### 4.3. Viral DNA Quantification

Total DNA was purified using the NZY Tissue gDNA Isolation kit (NZYtech, Lisbon, Portugal) following the manufacturer’s instructions. HSV-1 DNA levels were quantified by real-time qPCR using a CFX-384 Real-Time PCR System (Bio-Rad, Hercules, CA, USA) and a custom-designed TaqMan assay targeting the US12 viral gene (forward primer: 5′-CGTACGCGATGAGATCAATAAAAGG-3′; reverse primer: 5′-GCTCCGGGTCGTGCA-3′; TaqMan probe: 5′-AGGCGGCCAGAACC-3′). Viral DNA content was normalized to the amount of human genomic DNA using a predesigned TaqMan assay for the 18S rRNA gene (Hs9999991_s1; Applied Biosystems, Waltham, MA, USA). Data were analyzed using Bio-Rad CFX maestro 2.2. software. Quantification results are expressed as the number of HSV-1 DNA copies per ng of genomic DNA.

### 4.4. Immunofluorescence Analysis

#### 4.4.1. 2D Cultures

Cells grown on glass coverslips were fixed in 4% paraformaldehyde (PFA) and permeabilized with blocking solution containing 2% foetal calf serum and 0.2% Triton X-100 in phosphate buffer saline (PBS). Coverslips were then incubated with the corresponding primary antibodies, followed by Alexa Fluor-coupled secondary antibodies, both diluted in blocking solution (Table 1). Finally, nuclei were counterstained with 4,6-diamino-2-phenylindole (DAPI; Merck, Rahway, NJ, USA) in PBS and mounted on glass slides using Mowiol mounting medium (Sigma-Aldrich). All procedures were carried out at room temperature (RT) with samples protected from light.

#### 4.4.2. 3D Cultures

LUHMES 3D aggregates were collected in 1.5 mL tubes, and immunofluorescence analysis was performed as previously described [26]. Briefly, aggregates were washed once with cold PBS and fixed with 2% PFA for 1 h at 4 °C. After fixation, samples were permeabilized with blocking buffer containing 10% foetal calf serum, 1% bovine serum albumin (BSA) and 0.15% saponin in PBS for 1 h at 4 °C under gentle agitation. Primary antibody incubation was performed in 4-well or 24-well plates for 96 h at 4 °C under agitation, using antibodies diluted in blocking buffer. Then, samples were incubated with Alexa Fluor-coupled secondary antibodies for 72 h at 4 °C under the same conditions. Nuclei were counterstained with DAPI for 1 h at RT with agitation. Finally, after immunostaining, 3D LUHMES aggregates were cleared using RapiClear™ 1.49 (Sunjin Lab, Hsinchu City, Taiwan) to ensure uniform antibody penetration and optical transparency. Aggregates were mounted using 0.25 mm iSpacers (Sunjin Lab) adhered to microscope slides. Aggregates were placed within the iSpacers with minimal PBS, and the chamber was filled with RapiClear 1.49 before sealing with a coverslip. Slides were left to dry overnight at RT and stored at 4 °C in the dark until imaging.

#### 4.4.3. Fluorescence and Confocal Microscopy

Sample visualization was carried out using an inverted Axiovert 200M widefield microscope (ZEISS, Jena, Germany) coupled to a PCO edge 4.2 bi camera and a laser scanning ZEISS LSM900 confocal vertical system coupled to an Axio Imager 2 upright microscope (ZEISS). Images were acquired using 40× or 63× oil immersion objectives for cellular visualization and a 20× objective to capture complete 3D aggregates. For 3D cultures, images at 20× magnification were obtained from the central optical plane after acquiring a complete z-stack through the aggregate. In each experiment, 3–5 fields per condition (30–50 cells/field) were imaged, resulting in 150–400 cells analysed per experiment, and all experiments were repeated at least three times independently. Immunofluorescence images were acquired using Metamorph 7.10.5.476 or ZEN Blue 3.4 imaging software and subsequently processed with Fiji/ImageJ v1.54r and Adobe Photoshop 22.1.1.

### 4.5. Cell Lysates and Western Blot Analysis

Cell lysates were prepared by incubating samples in the radioimmunoprecipitation assay (RIPA) buffer (10 mM Tris-HCl pH 7.5, 50 mM NaCl, 1% Nonidet P-40, 0.2% sodium deoxycholate, 0.1% sodium dodecyl sulphate (SDS), and 1 mM EDTA) supplemented with protease (CompleteTM Mini, EDTA-free Protease Inhibitor Cocktail, Roche, Basel, Switzerland) and phosphatase (PhosSTOPTM, Roche) inhibitors. Protein concentrations in the cell lysates were determined by bicinchoninic acid assay (BCA, ThermoFisher Scientific) according to the manufacturer’s instructions. Equal amounts of protein were resolved by Laemmli discontinuous SDS–polyacrylamide gel electrophoresis (SDS-PAGE) and transferred onto nitrocellulose membranes. Membranes were blocked with either 3% BSA and 0.2% Tween 20 in PBS or 5% non-fat milk and 0.2% Tween 20 in PBS. Incubations with primary and peroxidase-coupled secondary antibodies (Table 1) were performed for 1 h at RT. Finally, protein bands were detected using enhanced chemiluminescence reagents (ECL, Amersham Biosciences, Amersham, UK) according to the manufacturer’s instructions. Densitometric quantification of protein bands was performed using Image Lab 6.0.1 software (Bio-Rad).

### 4.6. Quantification of mRNA Levels

mRNA expression levels were determined by RT-qPCR. Total RNA was isolated using the NZY Total RNA Isolation kit (NZYtech). Complementary DNA (cDNA) was synthesized from total RNA using the High-Capacity RNA-to-cDNA Kit (Applied Biosystems). cDNAs were amplified by PCR using primers specific for neural progenitor and neuronal genes (Table 2). Gene expression values were normalized to β-actin, used as a reference gene due to its stable expression across experimental conditions. Real-time qPCR assays were performed in a CFX-384 Real-Time PCR System (Bio-Rad), and data were analyzed using BioRad CFX maestro 2.2. software.

### 4.7. Secreted Aβ Measurements

Conditioned media from control and HSV-1-infected samples were assayed for human Aβ40 and Aβ42 levels using commercial sandwich enzyme-linked immunosorbent assay (ELISA) kits (Wako, Tokyo, Japan), following the manufacturer’s instructions. Culture media were first collected and inactivated by ultraviolet exposure. After centrifugation, supernatants were stored at −70 °C until use. Prior to analysis, samples were concentrated by lyophilization and resuspended in PBS. An 8-fold concentration was applied for LUHMES 2D cultures and a 4-fold concentration for 3D LUHMES aggregate media. Detection was based on a colorimetric reaction generated by the anti-Aβ detection antibody, and absorbance was measured at 450 nm using a Model 680 microplate reader (Bio-Rad). Final concentrations of Aβ40 and Aβ42 were expressed as picomoles per liter (pM) of culture medium.

### 4.8. Quantification of Lysosome Load

Lysosomal load was assessed using the acidotropic probe LTR (ThermoFisher Scientific), which freely diffuses across cell membranes and accumulates in acidic organelles. One hour before the end of the treatment period, cells were incubated with 0.5 µM LTR in culture medium for 1 h at 37 °C and subsequently washed with PBS. Cells were then lysed in RIPA buffer and centrifuged at 13,000× *g* for 10 min. Protein concentration in the lysates was determined using the BCA assay. LTR fluorescence intensity in the cell lysates was measured using a FLUOstar OPTIMA microplate reader (BMG LABTECH, Saitama, Japan) with excitation and emission wavelengths of 560 nm and 590 nm, respectively.

### 4.9. Cathepsin Activity Assays

The enzymatic activity of cathepsins was determined as previously described, with minor modifications [55]. Briefly, cells were lysed under gentle shaking in 50 mM sodium acetate buffer (pH 5.5) containing 0.1 M NaCl, 1 mM EDTA, and 0.2% Triton X-100. Lysates were clarified by centrifugation, and the supernatants were immediately used for the determination of proteolytic activity. A total of 25 µg (cathepsin D/E assay) or 100 µg (cathepsin S assay) of protein from each lysate was incubated for 30 min at 37 °C with the following fluorogenic substrates (Enzo Life Sciences, Farmingdale, NY, USA): Z-VVR-7-amino-4-methylcoumarin (Z-VVR-AMC; 20 µM), a specific substrate for cathepsin S, and Mca-GKPILFFRLK (Dnp)-D-Arg-NH_2_ (10 µM), a fluorogenic substrate for cathepsins D and E. Fluorescence resulting from substrate cleavage was measured using a Spark^®^ multimode microplate reader (Tecan, Männedorf, Switzerland) with excitation/emission wavelengths of 360/430 nm for cathepsin S substrate and 320/400 nm for the cathepsin D/E substrate.

### 4.10. Statistical Analysis

Statistical differences between groups were analyzed pairwise using a two-tailed Student’s *t*-test, or a One-sample *t*-test when data were expressed as relative values. ANOVA and multiple comparisons (Dunnet’s test) were applied to quantitative data obtained from ELISA. When needed, mixed-effects model was used instead of ANOVA. Statistical significance was set at *p* < 0.05 (*), *p* < 0.01 (**), *p* < 0.001 (***), and *p* < 0.0001 (****). The statistical analyses were performed using Microsoft Excel and GraphPad software 10.5.0.

## Figures and Tables

**Table 1:** List of antibodies used in Western blot (WB) and immunofluorescence (IF) analysis.

Target		Dilution			Reference	Manufacturer
		WB	IF			
			2D	3D		
Primary antibodies	NF200			1:1000	N4142	Sigma (St. Louis, MO, USA)
	βIII-Tub	1:10,000	1:1000	1:1000	ab18207	Abcam (Cambridge, UK)
	MAP2 (2A+2B)	1:250	1:250	1:500	M 1406	Sigma
	MAP2 (2A+2B+2C)	1:250			sc-74421	Santa Cruz (Dallas, TX, USA)
	NeuN		1:250		ab104224	Abcam
	Nestin	1:1000	1:250	1:500	656801	BioLegend (San Diego, CA, USA)
	APP	1:4000			MAB348	Millipore (Burlington, MA, USA)
	Total Tau	1:1000			NB100-82247	Bio-Techne (Abingdon, UK)
	P-Tau Thr205		1:100	1:250	44-738G	ThermoFisher Scientific (Waltham, MA, USA)
	P-Tau Ser422		1:100	1:250	44-764G	ThermoFisher Scientific
	P-Tau Ser202	1:250			ab9674	Sigma
	P-Tau Thr231	1:250			44-746G	ThermoFisher Scientific
	Aβ40		1:250	1:500	44-348A	ThermoFisher Scientific
	Aβ42		1:250	1:500	44-344	ThermoFisher Scientific
	ICP4	1:1000		1:1000	ab6514	Abcam
	ICP8	1:1000			sc-51906	Santa Cruz
	UL42	1:1000			sc-53329	Santa Cruz
	gC	1:3000	1:1000	1:1000	ab6509	Abcam
	gB/gD			1:1000	Provided by Dr. Tabares	---
	LC3/LC3-II	1:1000	1:250	1:500	L7543	Sigma
	Ki-67		1:500	1:500	ab16667	Abcam
	SOX2	1:1000			ab79351	Abcam
	GAPDH	1:1000			sc-51906	Santa Cruz
	α-tubulin	1:10,000			T5168	Sigma
Secondary antibodies	Anti-Mouse-POD	1:25,000			PI-2000	Vector (Newark, CA, USA)
	Anti-Rabbit-POD	1:25,000			GAR/IgG (H+L)/PO	Nordic (Susteren, The Netherlands
	Alexa-488 anti-mouse		1:1000	1:500	A-21202	ThermoFisher Scientific
	Alexa-555 anti-mouse		1:1000	1:500	A-31570	ThermoFisher Scientific
	Alexa-647 anti-mouse		1:1000	1:500	A-31571	ThermoFisher Scientific
	Alexa-488 anti-rabbit		1:1000	1:500	A-21206	ThermoFisher Scientific
	Alexa-555 anti-rabbit		1:1000	1:500	A-31572	ThermoFisher Scientific
	Alexa-647 anti-rabbit		1:1000	1:500	A-31573	ThermoFisher Scientific

**Table 2:** List of primers used in RT-qPCR analysis of LUHMES cells differentiation.

Genes *		Forward Primer	Reverse Primer
Proliferation	*MKI67*	5′-ATCGTCCCAGGTGGAAGAGTT-3′	5′-ATAGTAACCAGGCGTCTCGTGG-3′
Neuronal differentiation	*SYN1*	5′-TCAGACCTTCTACCCCAATCA-3′	5′-GTCCTGGAAGTCATGCTGGT-3′
	*SYP*	5′-CGAGGTCGAGTTCGAGTA CC-3′	5′-AATTCGGCTGACGAGGAGTA-3′
	*DRD2*	5′-GCCGGGTTGGCAATGATGCA-3′	5′-ACGGCGAGCATCCTGAACTT-3′
Reference	*ACTB*	5′-AGTGTGACGTGGACATCCGCAAAG-3′	5′-ATCCACATCTGCTGGAAGGTGGAC-3′ 5′;-GTCCACCTTCCAGCAGATGTGGAT-3′

* *MKI67* (Ki-67); *SYN1* (synapsin I); *SYP* (synaptophysin); *DRD2* (D2 dopamine receptor); *ACTB* (β-actin).

## Data Availability

The original contributions presented in this study are included in the article/Supplementary Materials. Further inquiries can be directed to the corresponding authors.

## Supplementary Materials

The following supporting information can be downloaded at: https://www.mdpi.com/article/10.3390/ijms27020642/s1.

## Abbreviations

2D	Two-dimensional
3D	Three-dimensional
AD	Alzheimer’s disease
APP	Amyloid precursor protein
Aβ	Beta-amyloid
BCA	Bicinchoninic acid assay
BSA	Bovine serum albumin
cDNA	Complementary DNA
CNS	Central nervous system
DAPI	4,6-diamino-2-phenylindole
ELISA	Sandwich enzyme-linked immunosorbent assay
hpi	Hours post-infection
iPSCs	Induced pluripotent stem cells
HSV-1	Herpes simplex virus type 1
LTR	LysoTracker Red
MAP2	Microtubule-associated protein 2
MOI	Multiplicities of infection
NeuN	Neuronal nuclei antigen
NF200	Neurofilament heavy chain
NFTs	Neurofibrillary tangles
PBS	Phosphate buffer saline
PFA	Paraformaldehyde
PFU	Plaque forming units
RIPA	Radioimmunoprecipitation assay
RT	Room temperature
SD	Standard deviation
SDS	Sodium dodecyl sulphate
SEM	Standard error of the mean
UL46-GFP	HSV-1 strain expressing GFP fused to the UL46 tegument protein
