# Supplementary figures and images for: Impact of HSV-1 Infection on Alzheimer’s Disease Neurodegeneration Markers: Insights from LUHMES 2D and 3D Neuronal Models
Source: Int J Mol Sci. 2026 Jan 8;27(2):642. doi: 10.3390/ijms27020642 (PMC12841366; doi:10.3390/ijms27020642)

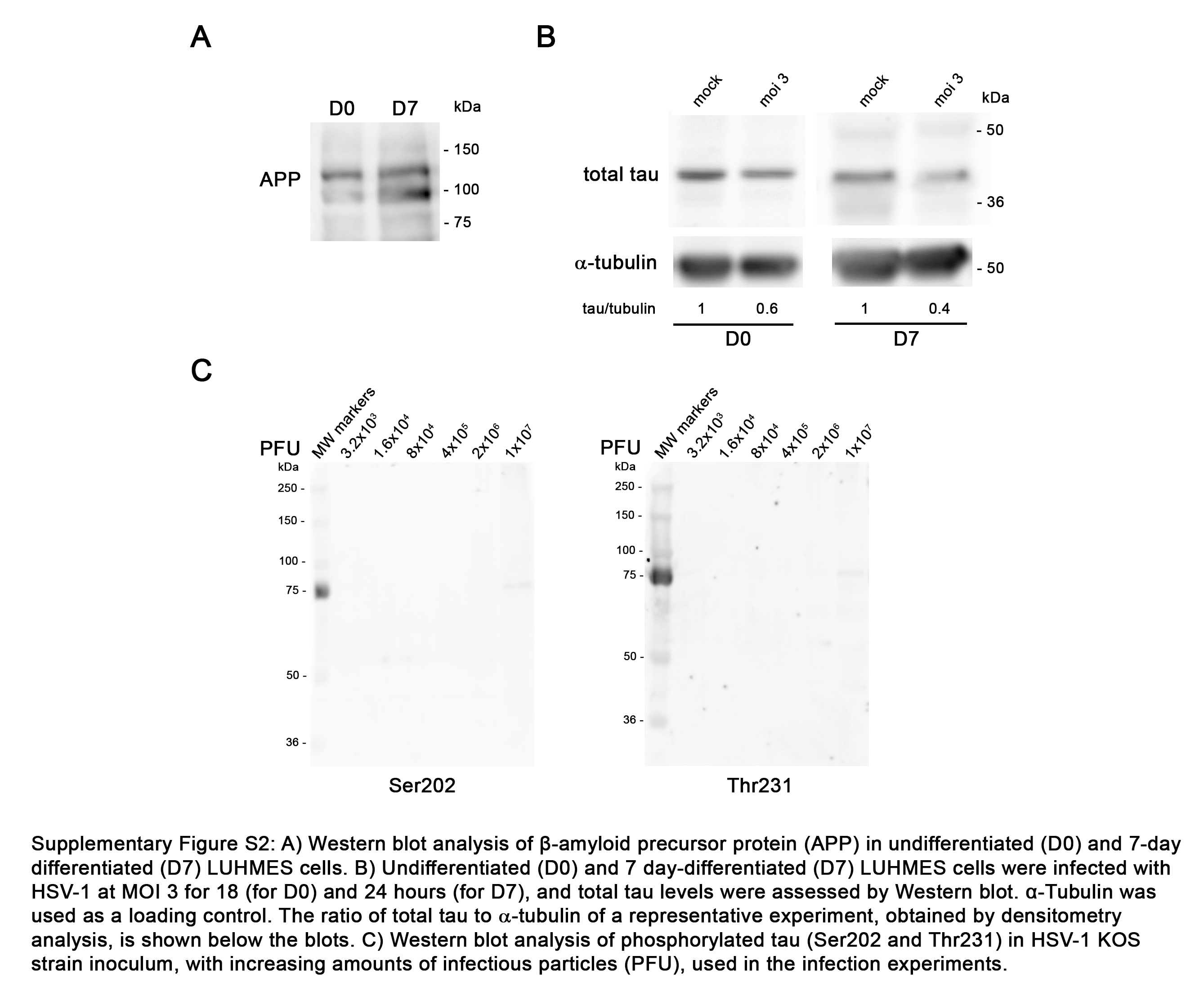

Supplement: Supplementary file 1 [file ijms-27-00642-s001.zip › Figure S2.tif]

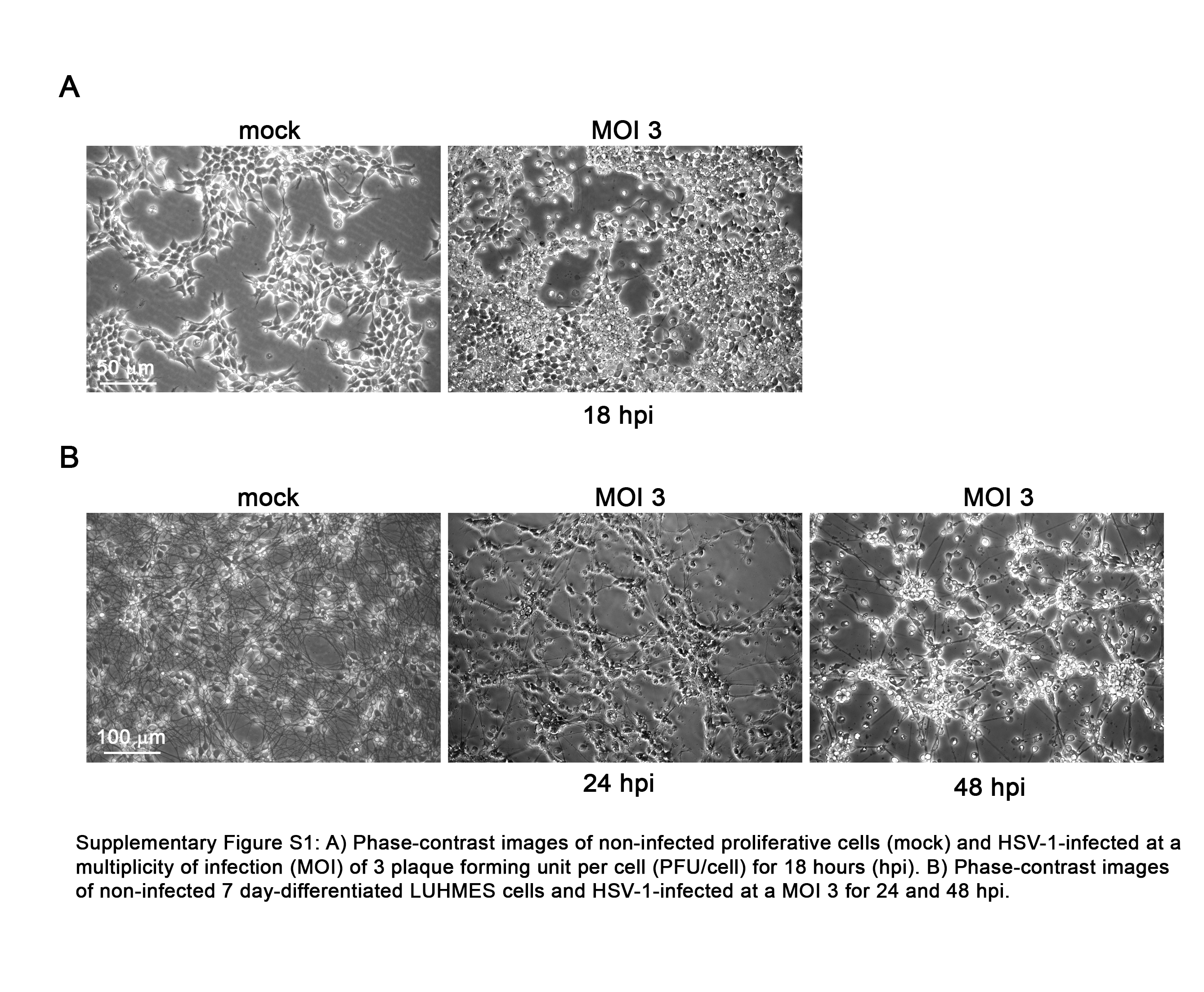

Supplement: Supplementary file 1 [file ijms-27-00642-s001.zip › Figure S1.tif]
